# Predicting 1-Year Trifecta Outcomes After High-Intensity Focused Ultrasound and Cryoablation for Low- and Intermediate-Risk Prostate Cancer

**DOI:** 10.3390/biomedicines14030716

**Published:** 2026-03-20

**Authors:** Umberto Anceschi, Antonio Tufano, Gabriele Tuderti, Riccardo Mastroianni, Simone D’Annunzio, Maria Consiglia Ferriero, Flavia Proietti, Lorenzo Capecchi, Giuseppe Spadaro, Maddalena Iori, Leonardo Misuraca, Franco Lugnani, Giuseppe Simone

**Affiliations:** 1 Department of Urology, IRCCS “Regina Elena” National Cancer Institute, 00144 Rome, Italy; 2 Department of Urology, San Carlo di Nancy Hospital, 00165 Rome, Italy; 3 Department of Maternal-Infant and Urological Sciences, “Sapienza” Rome University, Policlinico Umberto I Hospital, 00185 Rome, Italy

**Keywords:** HIFU, cryoablation, focal therapy, prostate cancer

## Abstract

**Objectives**: Focal therapy (FT) is increasingly employed in selected patients with localized prostate cancer (PCa), aiming to balance oncologic control with preservation of urinary and sexual function. Among the available energy sources, prostate gland cryoablation (PGC) and high-intensity focused ultrasound (HIFU) are the most widely adopted techniques. However, comparative outcome data remain limited, and standardized composite endpoints are still lacking in this domain. **Methods**: We conducted a prospective, single-center study on 163 consecutive patients treated with FT, either HIFU (*n* = 49) or PGC (*n* = 114), for clinically localized PCa between 2019 and 2024. The primary aim was to compare baseline, perioperative, oncologic, and functional outcomes at 1-year follow-up. The secondary objective was to identify predictors of trifecta achievement, defined as: (1) absence of treatment failure; (2) urinary continence (no pads or one safety pad/day); and (3) recovery of erectile function comparable to baseline. **Results**: HIFU was associated with shorter operative time (*p* = 0.04) but required longer catheterization (*p* < 0.001). Compared with HIFU, primary whole-gland cryoablation (PGC) showed a higher overall complication rate (*p* < 0.001), mostly grade I–II events. Median follow-up was shorter for HIFU (12 vs. 23 months, *p* < 0.001). Further, 1-year treatment failure occurred in 8.1% of HIFU cases and 8.7% of PGC cases (*p* = 0.96), although failure-free survival was comparable between groups (*p* = 0.89). Functional outcomes were similar, with no significant differences in continence or potency recovery, and trifecta rates were 38.9% (HIFU) vs. 37.4% (PGC; *p* = 0.355). On multivariable analysis, hypertension, lower PSA, higher baseline erectile function, and unilateral ablation independently predicted trifecta achievement. **Conclusions**: In this prospective comparison between HIFU and PGC, we observed similar trifecta achievement rates, with no significant differences in continence or erectile function recovery at 1 year. Although treatment failure was slightly more frequent after HIFU, overall outcomes support the functional safety and oncologic feasibility of both approaches in selected patients. These findings suggest that adopting a standardized composite endpoint may be clinically useful, even if further refinement and validation are still needed to capture the specific goals and nuances of focal therapy.

## 1. Introduction

Focal therapy (FT) has emerged as a promising treatment alternative for selected patients with localized prostate cancer (PCa), particularly in cases with low to intermediate oncologic risk [1,2]. By targeting the visible index lesion and sparing surrounding prostate tissue, FT aims to balance oncologic control with preservation of continence and sexual function [3,4]. Compared to whole-gland therapies such as radical prostatectomy or radiation therapy, focal approaches—most notably cryoablation and high-intensity focused ultrasound (HIFU)—have shown favorable safety profiles, low morbidity, and the potential for repeat treatments in case of recurrence, thereby preserving future therapeutic options [5,6,7].

Despite growing interest and widespread adoption in selected centers, the standardization of outcome metrics in FT remains an unresolved issue [8]. In radical prostatectomy, composite endpoints such as the trifecta and pentafecta are widely used to simultaneously capture surgical quality, oncologic control, and functional outcomes [9,10,11,12]. In contrast, focal therapy lacks a universally accepted composite framework, in part due to intrinsic differences in treatment objectives and the absence of clearly defined pathological surrogates such as surgical margins. Moreover, the oncologic component of the trifecta remains ill-defined in FT, where heterogeneous definitions of biochemical recurrence and variable follow-up strategies hinder uniform comparisons across studies [13,14]. This ambiguity has generated criticism, since the oncologic validity of trifecta models is often questioned by experts in the field, and no consensus currently exists regarding the optimal composite metrics to be applied to FT outcomes [13,15]. Nonetheless, the implementation of reproducible, multidimensional endpoints is essential to improve the quality of evidence, guide clinical decision-making, and facilitate comparisons between focal modalities [3,16].

To overcome these limitations, we conducted a prospective, single-center study comparing prostate gland cryoablation (PGC) and HIFU for the treatment of low- and intermediate-risk PCa, with the primary aim of evaluating one-year trifecta achievement using a standardized definition that includes oncologic control, continence, and sexual recovery. Secondary objectives included the analysis of treatment failure patterns and the identification of clinical predictors of trifecta success.

## 2. Materials and Methods

### 2.1. Study Design and Patient Selection

This is a prospective, single-center study based on the institutional focal therapy dataset of the IRCCS Regina Elena National Cancer Institute (Rome, Italy). From this dataset, we identified all consecutive patients who underwent focal therapy, either HIFU or PGC, for clinically localized prostate cancer between January 2019 and August 2024. The study was approved by the local institutional review board (Protocol Code: FT-PCA-1925), and written informed consent was obtained from all patients before treatment.

Eligible patients were required to have histologically confirmed, organ-confined prostate cancer with a prognostic Gleason Grade Group ≤ 2, a visible and well-characterized lesion on multiparametric magnetic resonance imaging (mpMRI), and a minimum follow-up of 12 months. Exclusion criteria included prior prostate cancer treatments, multifocal disease not suitable for focal therapy, incomplete follow-up data, or preoperative evidence of metastatic progression.

### 2.2. Treatment Modalities and Group Allocation

Patients were stratified into two treatment arms: Group A included those treated with HIFU, and Group B included those treated with PGC. Both whole-gland and partial ablations were performed, based on disease laterality and lesion characteristics on mpMRI. Treatment modality and ablation extent were decided by the multidisciplinary urologic-oncology team according to tumor location, prostate anatomy, and patient-specific factors.

### 2.3. Definition of Study Endpoints

The primary endpoint of the study was to compare baseline, perioperative, oncologic, and functional outcomes between patients treated with HIFU and those treated with PGC. Specifically, differences in complication rates, catheterization time, sexual and urinary recovery, and oncologic control at 1-year follow-up were assessed across treatment arms. The secondary endpoint was the identification of independent predictors of 1-year trifecta achievement through multivariable logistic regression analysis.

### 2.4. Definition of Trifecta

Trifecta achievement at 1 year was defined as the simultaneous fulfillment of the following criteria:

1. Oncologic control, intended as the absence of treatment failure (no need for salvage/re-treatment, no evidence of disease progression, and no metastases at 12 months).
2. Urinary continence, defined as the complete absence of pad use or use of one safety pad/day.
3. Sexual function recovery, defined as recovery of spontaneous or PDE5-I–assisted erections comparable to baseline status.

### 2.5. Data Collection and Variables

Collected variables included age, body mass index (BMI), ASA score, presence of hypertension or diabetes, preoperative PSA, prostate volume, mpMRI-derived PI-RADS score and lesion size, as well as pre-treatment erectile function assessed by the International Index of Erectile Function (IIEF-5). Biopsy characteristics included the number and percentage of positive cores. Perioperative data included operative time, catheterization duration, hospital stay, and postoperative complications graded according to the Clavien–Dindo classification. Follow-up assessments at 3, 6, and 12 months included PSA measurements, mpMRI imaging, and structured functional evaluations.

### 2.6. Statistical Analysis

Continuous variables were reported as medians with interquartile ranges (IQRs) and compared using the Mann–Whitney U test. Categorical variables were expressed as counts and percentages and analyzed using Pearson’s χ^2^ or Fisher’s exact test, as appropriate. Kaplan–Meier analysis was used to evaluate time to treatment failure and recovery of functional domains; intergroup comparisons were performed with the log-rank test. Multivariable logistic regression was performed to identify independent predictors of trifecta achievement at 1 year. A two-sided *p*-value < 0.05 was considered statistically significant. Analyses were performed using SPSS Statistics, version 28.0 (IBM Corp., Armonk, NY, USA).

## 3. Results

A total of 163 patients met the inclusion criteria and were analyzed: 49 (30.1%) underwent HIFU, while 114 (69.9%) were treated with PGC. The two cohorts were similar in terms of age (median: 74 years for both; *p* = 0.126), BMI (median: 25 vs. 26.7 kg/m^2^; *p* = 0.634), prostate volume (*p* = 0.910), preoperative PSA levels (*p* = 0.334), and erectile function measured by IIEF-5 (*p* = 0.567). The number of positive biopsy cores and the percentage of involvement were comparable (*p* = 0.08 and *p* = 0.316, respectively). Gleason Grade Groups and PI-RADS scores were evenly distributed between the two groups (each *p* > 0.07). Notably, a higher proportion of patients in the PGC group had ASA scores of 3–4 (43% vs. 18.4%; *p* = 0.03) (Table 1).

Intraoperatively, median operative time was shorter for HIFU compared to PGC (45 vs. 55 min; *p* = 0.04) (Table 2). Conversely, the duration of catheterization was significantly longer in the PGC group (15 vs. 7 days; *p* < 0.001), despite a shorter hospital stay (2 vs. 3 days; *p* < 0.001). Complication rates were higher after PGC (31.5% vs. 20.4%; *p* < 0.001). Most events were classified as Clavien–Dindo grade I–II, with hematuria and orchitis being the most frequent. Only one severe complication (recto-vesical fistula) occurred in the HIFU group and one urethral fistula in the PGC group (*p* = 0.455).

**Figure 1:**
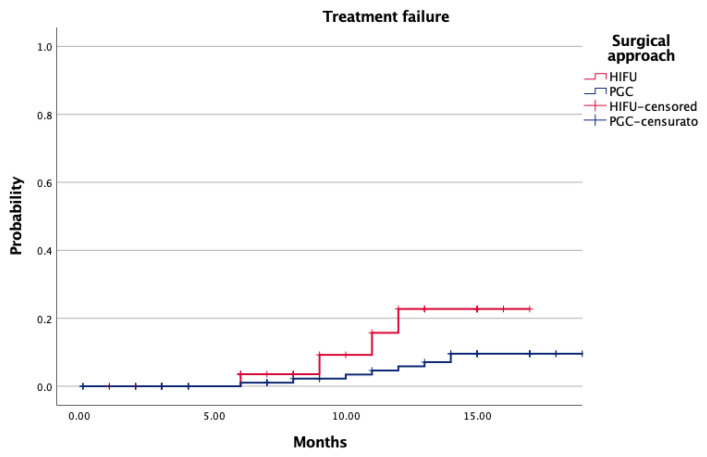
Probability of treatment failure according to surgical approach.

Median follow-up was significantly shorter for HIFU-treated patients (12 vs. 23 months; *p* < 0.001). Additionally, 1-year treatment failure occurred in 8.1% of HIFU and 8.7% of PGC (*p* = 0.96). Most recurrences were in-field or biochemical; out-of-field failures were rare and occurred only in the PGC group (2.6%; *p* = 0.91). The proportion of patients requiring salvage or systemic treatment remained low across both cohorts (Table 3). Kaplan–Meier analysis revealed no significant difference in failure-free survival between the two groups (Figure 1, *p* = 0.08). 

**Figure 2:**
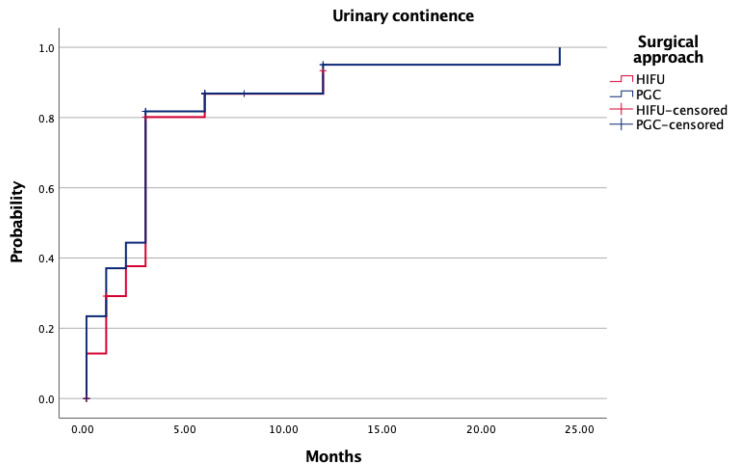
Probability of continence recovery according to surgical approach.

**Figure 3:**
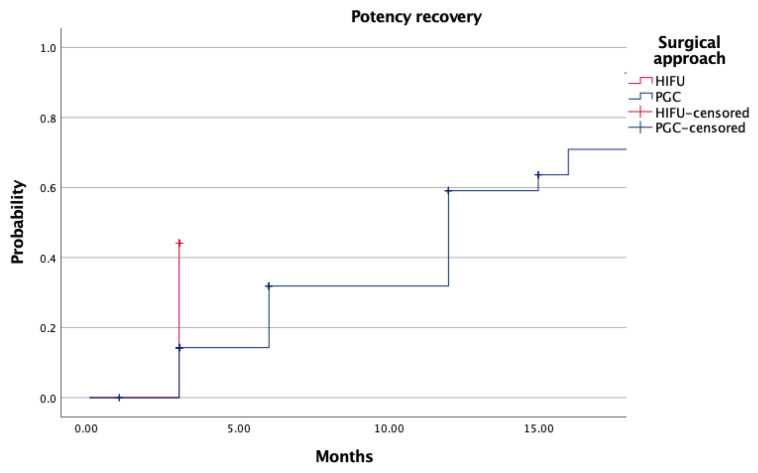
Probability of potency recovery according to surgical approach.

Functional outcomes at 12 months were similar between arms. Urinary continence rates exceeded 88% in both groups (*p* = 0.76), and sexual function recovery—defined as spontaneous or PDE5-I–assisted erections—was achieved in 44.8% (HIFU) and 37.5% (PGC) of patients (*p* = 0.497) (Table 4). Kaplan–Meier curves for continence and potency recovery confirmed equivalent recovery dynamics (Figure 2, *p* = 0.89; Figure 3, *p* = 0.78). At 12 months, trifecta achievement was documented in 38.9% of HIFU patients and 37.4% of those treated with PGC (*p* = 0.355) (Table 4).

Multivariable logistic regression identified hypertension (OR: 3.02; 95% CI: 1.16–7.86; *p* = 0.02), lower preoperative PSA (OR: 0.84; 95% CI: 0.73–0.96; *p* = 0.01), higher baseline erectile function (OR: 0.32; 95% CI: 0.12–0.85; *p* = 0.02), and unilateral ablation (OR: 5.3; 95% CI: 1.75–16.05; *p* = 0.03) as independent predictors of trifecta achievement (Table 5). ASA score, BMI, age, diabetes, prostate volume, and surgical approach (HIFU vs. PGC) did not emerge as significant predictors. 

## 4. Discussion

The integration of focal therapy (FT) into the contemporary urologic armamentarium for localized PCa responds to a growing clinical demand: maximizing oncologic control while preserving urinary and sexual function. This prospective comparative analysis of HIFU versus PGC aims to critically appraise this equilibrium, employing a tailored trifecta model specific to FT. By doing so, it contributes to the evolving discourse around composite outcome metrics in a field where terminological and methodological standardization remains elusive.

Baseline characteristics across the two groups were broadly balanced, with the only significant difference emerging in ASA score distribution—patients undergoing cryoablation were more frequently classified as ASA 3–4 (43% vs. 18.4%, *p* = 0.03; Table 1). This disparity likely reflects clinical prudence, as cryoablation, which generally entails shorter procedural time and minimal anesthesia requirements, is often preferred in older or more comorbid patients [17]. Nevertheless, no substantial differences were observed in other baseline variables, including age, body mass index, PSA levels, erectile function (IIEF-5), prostate volume, or biopsy metrics. This internal comparability strengthens the interpretive validity of our comparative findings.

Perioperative outcomes revealed distinct procedural profiles. HIFU was associated with shorter operative times (*p* = 0.04), but significantly longer catheterization duration (15 vs. 7 days, *p* < 0.001) and hospital stay (3 vs. 2 days, *p* < 0.001; Table 2). These differences are attributable to the distinct ablative mechanisms of the two modalities: tissue cavitation and edema in HIFU may necessitate prolonged drainage, while cryoablation often allows faster catheter removal. Importantly, overall complication rates were higher in the PGC group (31.5% vs. 20.4%, *p* < 0.001), especially for minor events such as orchitis, hematuria, and transient fever. This trend aligns with existing literature, where cryoablation is associated with an increased incidence of local inflammatory responses and urethral irritation, particularly in whole-gland settings. Severe complications were rare and comparable between groups [18,19].

Oncologic control, measured by treatment failure at one year, was slightly better in the PGC group (2.3% vs. 8.1%, *p* = 0.04; Table 3), yet this difference may reflect lead-time bias, given the significantly longer follow-up in the PGC cohort (23 vs. 12 months, *p* < 0.001). Kaplan–Meier curves (Figure 1) showed no significant difference in time to failure (*p* = 0.08), underscoring the importance of cautious interpretation when comparing cohorts with asymmetric surveillance durations. Salvage therapy and systemic progression were rare in both arms, reaffirming the short-term oncologic feasibility of FT in selected patients.

Functional outcomes were satisfactory and similar between groups. Urinary continence at one year exceeded 88% in both arms (*p* = 0.76), while potency recovery—either spontaneous or pharmacologically assisted—reached 44.8% in the HIFU group and 37.5% in the PGC group (*p* = 0.497). Recovery dynamics over time did not differ significantly, reinforcing the functional safety profile of both techniques. These findings are especially relevant given the increasing demand for minimally morbid interventions in aging, sexually active patients [20,21].

Moreover, our results are consistent with the systematic review and meta-analysis published in 2018 [22], which reported comparable oncologic and functional outcomes between HIFU and cryotherapy in patients with localized prostate cancer. That analysis highlighted the absence of clear superiority of one modality over the other, supporting the interpretation that both techniques represent feasible and safe focal treatment options in appropriately selected patients.

Trifecta achievement, defined as simultaneous oncologic control, continence, and erectile function, was comparable between groups (38.9% vs. 37.4%, *p* = 0.355). This equilibrium underscores the feasibility of FT in meeting multidimensional patient expectations. However, it also highlights the need for more nuanced definitions. Unlike radical prostatectomy, where trifecta and pentafecta metrics are well-established, FT lacks a universally accepted composite outcome model [23]. The ambiguity surrounding oncologic endpoints (e.g., role of margins, definition of recurrence) continues to be debated among experts, and no formal consensus panel has yet validated a standard trifecta construct in this domain [23].

In our cohort, logistic regression identified hypertension (OR 3.02, *p* = 0.02), lower preoperative PSA (OR 0.84, *p* = 0.01), preserved erectile function at baseline (IIEF-5 > 21; OR 0.32, *p* = 0.02), and bilateral ablation (OR 5.3, *p* = 0.03) as independent predictors of trifecta achievement. These findings suggest that preoperative functional reserve and adequate oncologic targeting are key drivers of FT success. Interestingly, neither age nor ASA score remained significant at multivariable level, indicating that properly selected comorbid patients may still derive substantial benefit from FT.

Nonetheless, our study is not devoid of limitations. The non-randomized, observational nature of the dataset entails an intrinsic risk of selection bias. Although prospectively maintained, the cohort was not propensity-matched, and treatment allocation may reflect unmeasured confounders. The follow-up duration was substantially shorter in the HIFU arm, limiting the interpretability of oncologic comparisons. Trifecta criteria, although deliberately constructed, remain institution-specific and unvalidated externally. Functional outcomes were patient-reported, with all the inherent risk of underestimation or misclassification. Moreover, a potential limitation of the present study is the temporal imbalance between treatment cohorts, with patients treated with HIFU undergoing intervention more recently than those treated with cryoablation. This may introduce temporal bias related to procedural learning curves, ongoing technical refinements, improvements in imaging and perioperative management, and evolving patient selection criteria over time. Accordingly, differences in outcomes between groups should be interpreted with caution, as they may partly reflect temporal changes in clinical practice rather than treatment-specific effects. Furthermore, imaging interpretation and biopsy strategy were not centrally reviewed, potentially introducing heterogeneity in lesion characterization. An additional limitation is the imbalance in sample size between treatment groups, with fewer patients undergoing HIFU compared with cryoablation, which may influence statistical power and limit the ability to detect smaller between-group differences. Lastly, although regression modeling was performed to identify predictors, the relatively low number of events may limit the robustness of analyses.

Despite these limitations, our study offers several contributions to the current landscape of FT. It provides real-world comparative data between two widely adopted ablative modalities, offers a reproducible composite outcome metric, and identifies patient-specific factors predictive of clinical success. These findings may assist clinicians in refining patient selection and preoperative counseling, while contributing to the broader effort to harmonize FT endpoints across centers, including clear oncologic surrogates, long-term outcomes, quality-of-life metrics, and retreatment strategies.

## 5. Conclusions

Focal therapy represents a maturing paradigm for selected cases of localized prostate cancer, offering a valuable balance between cancer control and functional preservation. Our findings, based on a comparative analysis of HIFU and PGC, support the feasibility of adopting a composite endpoint such as trifecta, although its oncologic consistency remains under scrutiny. The identification of independent clinical predictors may assist in refining patient selection and strengthening preoperative counseling. Future prospective efforts should aim to harmonize outcome definitions and validate clinically meaningful endpoints in this emerging field.

## Figures and Tables

**Table 1:** Baseline characteristics of patients who underwent HIFU and Cryoablation.

Variable, *n*, %	HIFU *n* = 49 (30.1%)	Cryoablation *n* = 114 (69.9%)	*p*-Value
Age (years, median, IQR)	74 (67.5–75)	74 (70–76)	0.12
BMI (Kg/m^2^, median, IQR	25 (23–28)	26.7 (24.1–29)	0.63
ASA score (*n*, %)			**0.03**
1–2	40 (81.6%)	65 (57%)	
3–4	9 (18.4%)	49 (43%)	
Preoperative PSA (ng/mL, median, IQR)	7 (4.8–9.8)	7.5 (5.13–9.9)	0.33
Preoperative IIEF-5 (media, IQR)	13.5 (4.5–21)	11 (3–19)	0.56
Prostate Volume (cm^3^, median, IQR)	44 (30.6–65.2)	50.5 (32–63.7)	0.91
Total Biopsy cores (*n*, median, IQR)	15 (10–20)	16 (12–20)	0.86
Total Systematic Biopsies (*n*, median, IQR)	15 (10–20)	16 (12–20)	0.53
Total targeted biopsies (*n*, median, IQR)	3 (2–3)	3 (2–3)	0.89
Total positive cores (*n*, median, IQR)	4 (2–5)	4 (2–6)	0.08
% positive cores (*n*, median, IQR)	26.3 (17.6–40)	25.6 (14.6–42.8)	0.31
Year of procedure (*n*, %)			-
2019	-	-	
2020	-	-	
2021	-	31 (27.1%)	
2022	-	44 (38.6%)	
2023	20 (40.8%)	20 (17.6%)	
2024	29 (59.1%)	19 (16.7%)	
PIRADS score (*n*, %)			0.25
2	3 (6.1%)	9 (7.8%)	
3	13 (26.5%)	14 (12.2%)	
4	27 (55.2%)	70 (61.5%)	
5	6 (12.2%)	21 (18.5%)	
PIRADS area size (mm, median, IQR)	10 (7.5–14)	12 (8–15)	0.90
Biopsy Gleason Grade Group (*n*, %)			0.07
ISUP Group 1	25 (51%)	41 (36%)	
ISUP Group 2	24 (49%)	73 (64%)	
Approach (*n*, %)			0.27
Emigland	32 (65.3%)	64 (56.1%)	
Whole-Gland	17 (34.7%)	50 (43.9%)	

HIFU, high-intensity focused ultrasound; BMI, body mass index; ASA, American Society of Anesthesiologists; PSA, prostate specific antigen; IQR, interquartile range; IIEF-5, International Index of Erectile Function-5; ISUP, International Society of Urological Pathology. Bold means statistically significant result.

**Table 2:** Perioperative outcomes.

Variable	HIFU *n* = 49 (30.1%)	Cryoablation *n* = 114 (69.9%)	*p*-Value
Operative time (minutes, median, IQR)	45 (35–62)	55 (40–65)	**0.04**
Length of hospital stay (days, median, IQR)	3 (3–3)	2 (2–3)	**<0.001**
Time to catheter removal (days, median, IQR)	7 (7–7)	15 (15–15)	**<0.001**
Any complication	10 (20.4%)	36 (31.5%)	**<0.001**
Total number of complications			
1st complication	9 (18.3%)	31 (27.1%)	**0.048**
2nd complication	1 (2.1%)	5 (4.4%)	0.084
Time to 2nd complication (months, median, IQR)	1 (1–1)	2 (1–5)	0.432
Severe complications (*n*, %)	1 (2.1%)	1 (0.87%)	0.455
Clavien Dindo classification (*n*, detail, %)			**0.048**
1–2	9 (18.4%) (5 Orchitis, 1 Fever, 3 Hematuria)	35 (30.7%) (5 Hematuria, 17 Orchitis, 8, Fever, 5 Acute Urinary Retention)	
3–5	1 (2.1%) (Recto-vesical fistula)	1 (0.87%) (Urethral fistula)	

HIFU, high-intensity focused ultrasound; IQR, inter quartile range. Bold means statistically significant result.

**Table 3:** Oncologic outcomes.

Variable	HIFU *n* = 49 (30.1%)	Cryoablation *n* = 114 (69.9%)	*p*-Value
Follow-up months (months, median, IQR)	12 (10–15)	23 (11–30)	**<0.001**
Neoadjuvant ADT therapy (*n*, %)	3 (6.1%)	5 (4.38%)	0.416
Time to treatment failure (months, median, IQR)	8 (4–13)	20 (8–29)	0.89
Type of treatment failure (*n*, %)			0.91
In-field recurrence/Biochemical recurrence	4 (8.1%)	7 (6.1%)	
Out-of-field recurrence	-	3 (2.6%)	
Treatment failure: liberal definition (=rebiopsy for rising PSA, adjuvant radiotherapy, PSA > 0.1)			0.96
1-year treatment failure (*n*, %)	4 (8.1%)	10 (8.7%)	
Need for systemic treatment (*n*, %)			0.618
Systemic treatment at 24 months	1 (2%)	5 (2.6%)	
Adjuvant ADT (*n*, %)	-	2 (1.75%)	-
Salvage therapy (*n*, %)			0.98
Repeat PGC/HIFU	2 (4%)	4 (3.5%)	
Radical Prostatectomy	-	-	
Radiation therapy	-	-	
Radiation therapy and ADT	-	-	
ADT	2 (4%)	3 (2.6%)	
Metastatic disease (*n*, %)			-
24 months	-	3 (2.63%)	

IQR, interquartile range; ADT, androgen deprivation therapy; PSA: prostate-specific antigen; PGC, prostate gland cryoablation; HIFU, high-intensity focused ultrasound. Bold means statistically significant result.

**Table 4:** Functional outcomes.

Variable	HIFU *n* = 49 (30.1%)	Cryoablation *n* = 114 (69.9%)	*p*-Value
1-yr urinary continence (%)	89%	88.6%	0.76
1-yr Sexual recovery details (detail %)	44.8%	37.5%	0.497
Potency without drugs/device	19 (38.7%)	16 (14.1%)	
With PDE5-I	3 (6.1%)	27 (23.7%)	
With PGE-1	-	-	
No erections	27 (55.2%)	71 (62.2%)	
1-yr treatment failure (%)	8.1%	2.3%	**0.04**
1-yr Trifecta (defined as simultaneous cancer control, continence recovery and sexual recovery) (%)	38.9%	37.4%	0.355

HIFU, high intensity focused ultrasound; PDE5-I, type 5 inhibitors; PGE-1, Prostaglandin E1. Bold means statistically significant result.

**Table 5:** Logistic regression analysis for identifying predictors of 1-yr trifecta.

Variable	Univariable Analysis				Multivariable Analysis			
	OR	95% CI			OR	95% CI		
		Lower	Higher	*p*-Value		Lower	Higher	*p*-Value
Age at surgery	0.94	0.88	1.003	0.06	-	-	-	-
ASA score coded (3–4 vs. 1–2)	4.14	1.5	11.4	0.006	1.41	0.39	5.08	0.594
BMI	0.93	0.83	1.03	0.194	-	-	-	-
Hypertension (yes vs. no)	3.52	1.64	7.55	0.001	3.02	1.16	7.86	0.02
Diabetes (yes vs. no)	1.66	0.58	4.74	0.338	-	-	-	-
Preoperative PSA (ng/mL)	0.87	0.78	0.96	0.019	0.84	0.73	0.96	0.01
Prostate Volume (mpMRI-estimated)	0.99	0.98	1.01	0.634	-	-	-	-
PIRADS area size (mpMRI-estimated)	0.96	0.88	1.04	0.336	-	-	-	-
Preoperative IIEF-5 (>21 vs. >21)	0.29	0.12	0.68	0.004	0.32	0.12	0.85	0.02
Type of focal therapy (bilateral vs. unilateral)	4.27	1.74	10.5	0.002	5.3	1.75	16.05	0.03
Surgical approach (HIFU vs. Cryoablation)	0.84	0.38	1.89	0.687	-	-	-	-

ASA, American Society of Anesthesiologists; BMI, body mass index; PSA, prostate-specific antigen; mpMRI, multiparametric magnetic resonance imaging; IIEF-5, International Index of Erectile Function-5; HIFU, high intensity focused ultrasound, OR, odds ratio.

## Data Availability

The data presented in this study are available in this article.

## Abbreviations

The following abbreviations are used in this manuscript:

FT	Focal therapy
PCa	prostate cancer
PGC	prostate gland cryoablation
HIFU	high-intensity focused
